# Analytical Performance and Greenness Evaluation of Five Multi-Level Design Models Utilized for Impurity Profiling of Favipiravir, a Promising COVID-19 Antiviral Drug

**DOI:** 10.3390/molecules27123658

**Published:** 2022-06-07

**Authors:** Adel Ehab Ibrahim, Yasmine Ahmed Sharaf, Sami El Deeb, Rania Adel Sayed

**Affiliations:** 1 Natural and Medical Sciences Research Center, University of Nizwa, P.O. Box 33, Birkat Al Mauz, Nizwa 616, Oman; adel@unizwa.edu.om; 2 Analytical Chemistry Department, Faculty of Pharmacy, Port-Said University, Port Fuad 42526, Egypt; 3 Analytical Chemistry Department, Faculty of Pharmacy, Zagazig University, Zagazig 44519, Egypt; yasminesharaf2009eg@gmail.com (Y.A.S.); raniaadelsayed@gmail.com (R.A.S.); 4 Institute of Medicinal and Pharmaceutical Chemistry, Technische Universitaet Braunschweig, 38106 Braunschweig, Germany

**Keywords:** analytical greenness, COVID-19, favipiravir, impurity profiling, multi-level design

## Abstract

In 2018, the discovery of carcinogenic nitrosamine process related impurities (PRIs) in a group of widely used drugs led to the recall and complete withdrawal of several medications that were consumed for a long time, unaware of the presence of these genotoxic PRIs. Since then, PRIs that arise during the manufacturing process of the active pharmaceutical ingredients (APIs), together with their degradation impurities, have gained the attention of analytical chemistry researchers. In 2020, favipiravir (FVR) was found to have an effective antiviral activity against the SARS-COVID-19 virus. Therefore, it was included in the COVID-19 treatment protocols and was consequently globally manufactured at large-scales during the pandemic. There is information indigence about FVR impurity profiling, and until now, no method has been reported for the simultaneous determination of FVR together with its PRIs. In this study, five advanced multi-level design models were developed and validated for the simultaneous determination of FVR and two PRIs, namely; (6-chloro-3-hydroxypyrazine-2-carboxamide) and (3,6-dichloro-pyrazine-2-carbonitrile). The five developed models were classical least square (CLS), principal component regression (PCR), partial least squares (PLS), genetic algorithm-partial least squares (GA-PLS), and artificial neural networks (ANN). Five concentration levels of each compound, chosen according to the linearity range of the target analytes, were used to construct a five-level, three-factor chemometric design, giving rise to twenty-five mixtures. The models resolved the strong spectral overlap in the UV-spectra of the FVR and its PRIs. The PCR and PLS models exhibited the best performances, while PLS proved the highest sensitivity relative to the other models.

## 1. Introduction

The coronavirus pandemic swept over the world at the beginning of 2020 and altered the economy, scientific communication, and the overall nature of people’s lives. In March 2020, the World Health Organization (WHO) officially declared the COVID-19 outbreak as a global pandemic [1]. Despite the international efforts to decrease its spread, COVID-19 had spread to more than 210 countries. Up until the writing of this proposed manuscript, which was December 2021, COVID-19 has caused about five million deaths according to the official WHO COVID-19 Dashboard [2].

Relative to the majority of COVID-19 diagnosed cases, about 16% suffered from severe acute respiratory syndrome (SARS) accompanied by a hyper inflammatory phase, leading to multi-organ system failure [3,4]. Therefore, worldwide health care systems have born the great burden of developing strategies for the control of COVID-19 transmission and treatment, especially under the unavailability of sufficient information about this severe illness. All of the developed therapeutic strategies were based on potent antiviral drug administration together with self-quarantine. Such patient quarantine during treatment strategies proved to present an essential role in the suppression of the outbreak peak. Researchers have strived to develop viral vaccines or anti-viral drugs to restrain COVID-19 progression. The approval process of any newly developed human drug is very complex and needs a long time to state the safety data, efficacy, and potential risks. Therefore, to avoid the long time interval required to profile the safety of new drugs, the fastest way was to test the effectiveness of previously FDA approved antiviral drugs against SARS-COVID-19 infections. Several clinical trials were performed on the efficacy of some approved antiviral drugs on the SARS-COVID-19 virus including remdesivir, the lopinavir and ritonavir combination, tocilizumab, and favipiravir (FVR) [5].

**Figure 1:**
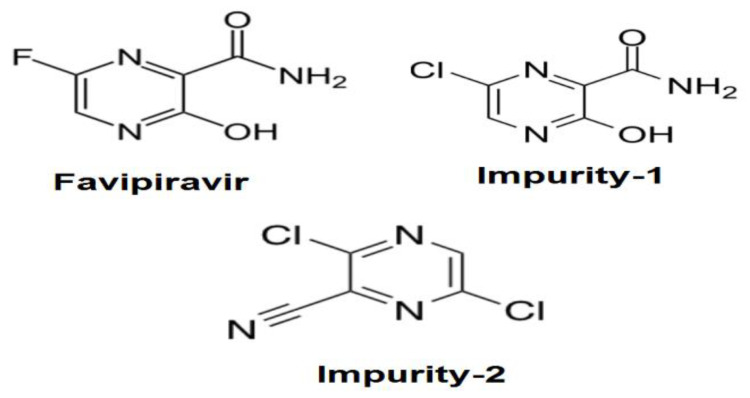
The chemical structures of FAV, impurity-1, and impurity-2.

FVR is an oral broad spectrum inhibitor of the RNA-dependent RNA polymerase (RdRp) found in the core of coronavirus and Nsp12-polymerase, which is very important in the life cycle of coronavirus, is an effective target for therapeutic interventions [6]. FVR (6-fluoro-3-oxo-3,4-dihydropyrazine-2-carboxamide) is also known as T-705 (the chemical structure is presented in Figure 1). FVR also has a potent inhibitory activity against influenza A, B, and C viruses in vitro and in vivo. FVR was approved for the new and reemerging influenza pandemic in Japan, and it has a well-known safety profile. Therefore, it has been clinically used in the treatment of COVID-19 [7].

In vitro studies demonstrated that FVR has an effective role in the COVID-19 treatment protocol with several advantages within a safe therapeutic dose [3]. First, being manufactured as an oral formulation, FVR therapy fulfills the needs of patients with mild to moderate COVID-19 infection, which can mostly be treated on an outpatient basis. Furthermore, FVR was stated as one of the most promising antiviral drugs due to its remarkable results regarding viral load reduction as well as an improvement in the radiological and clinical outcomes in COVID-19 patients. In addition, it is an advantageous medication regarding its mechanism of action, strength of the preclinical stage results, reassuring human safety data, bioavailability, good progress of cases, and manufacturing certainty [3]. Hence, it has been approved for the treatment of COVID-19 infections in many countries such as Turkey, Japan, India, Russia, the KSA, Italy, and Egypt [8].

Meanwhile, drug impurities represent an important issue during drug manufacturing. Impurities are formed during the manufacturing, formulation, and/or storage due to the drug decomposition. Most of these impurities have harmful effects either on the drug’s efficacy or even on its safety profile [9]. Regulatory bodies have stipulated that impurities that are above the critical concentration levels must be detected. The detection and quantification of PRIs that might be found within the active pharmaceutical ingredients (APIs) have occupied the minds of analytical chemistry researchers for years. The development of analytical methods for the quantitation and resolution of APIs in the presence of structurally analogous impurities is a great challenge, especially in cases where these impurities have similar chemical, physical, and spectral characteristics.

Studies have demonstrated that some of FVR’s structurally analogous impurities may lead to elevation in blood uric acid, resulting in hyperuricemia, especially in patients suffering from renal complications [10]. On the other hand, other impurities were found to be effective against other viral infections [10]. Therefore, there is a heavy demand to develop analytical methods for the simultaneous determination of FVR in the presence of its process-related impurities, especially given that there is an information indigence in the analytical data of these impurities.

In the proposed research, two important FVR processing impurities were studied. 6-Chloro-3-hydroxypyrazine-2-carboxamide (Impurity-1) and 3,6-dichloro-pyrazine-2-carbonitrile (Impurity-2) (the chemical structures are presented in Figure 1). FVR was synthesized from 2-aminopyrazine as the starting material. During the synthesis pathway, Impurity-1 was formed through the chlorination of the pyrazine ring in the intermediates [11]. Impurity-2 was found in 77% of the yield after FVR synthesis from 3-hydroxypyrazine-2-carboxamide, followed by nitration, producing the nitro compound. The nitro compound could be subjected to the displacement of the hydroxyl and nitro groups with chlorine and amide dehydration to nitrile, giving rise to Impurity-2 [12].

From this perspective, there is a persistent need for the simultaneous development of sensitive analytical methods for FVR determination with its process-related impurities. A review of the literature revealed few analytical methodologies that were reported for FVR determination using chromatographic techniques [13,14,15,16,17,18,19,20,21,22,23,24,25]. Three spectrofluorimetric methods [19,26,27] and two electrochemical methods [28,29] were reported for the determination of FVR. All of the mentioned methodologies were only reported for the determination of FVR alone either in plasma or as a pharmaceutical preparation. To the best of our knowledge, only two LC methods have been reported to be stable in indicating for the determination of FVR in the presence of degradation impurities, however, these did not consider the FVR process related impurities (PRIs) [30,31]. The FVR monograph has not yet been officially listed in any pharmacopoeia. The proposed PRIs, as indicated by the manufacturer’s synthetic pathway and batch analysis records [32], were determined by HPLC using the RP-C18 column and gradient program for a mobile phase composition that lasted for 90 min per run.

No paper has reported methods for the simultaneous determination of FVR together with its PRIs. The aim of this work was to develop five novel multi-level design models for the simultaneous determination of FVR and two of its process related impurities. The developed models were classical least square (CLS), principal component regression (PCR), partial least squares (PLS), genetic algorithm-partial least squares (GA-PLS), and artificial neural networks (ANN). The developed models were utilized to resolve the severe overlapped spectra of FVR and its studied process related impurities. A comparative study was then performed regarding the performances of the developed models in the impurity profiling of FVR.

## 2. Results

**Figure 2:**
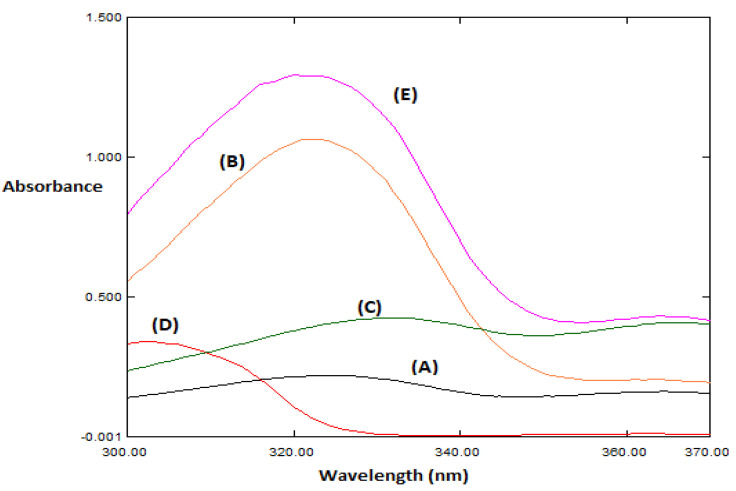
The absorption spectra of (A,B) 3 and 20 µg mL^−1^ FVR, respectively, (C) 3 µg mL^−1^ Impurity-1, (D) 3 µg mL^−1^ impurity-2; and (E) mixture of 20 µg mL^−1^ FVR, 3 µg mL^−1^ Impurity-1, and 3 µg mL^−1^ Impurity-2 in methanol.

The UV-spectroscopic scan of FVR and the two impurities under study revealed similar spectra. Severe overlapping was observed between the three spectra and seriously handicapped their direct determination (Figure 2). Univariate calibration methods could not resolve this severe spectral similarity and overlap. Multivariate models are useful in complex spectral analysis due to the inclusion of many spectral wavelengths instead of a single one, resulting in an improvement in the predictive ability and precision of the models [33]. Thus, five multivariate multi-level chemometric models (CLS, PCR, PLS, GA-PLS, and ANN) were useful in this mixture resolution.

### 2.1. Method Validation

#### 2.1.1. Calibration Matrix Design

The multicomponent design quality depends on the spectral zone selection and the used spectral mode. A calibration matrix of five levels and three factors was designed through the preparation of twenty-five mixtures containing different ratios from the three compounds so that each compound concentration has to be orthogonal to the other two compounds in the mixtures to collect the maximum information about the mixtures’ spectra. Twelve samples were utilized as a training set to build the calibration models, and the other thirteen samples were used as a validation set (see Section 3.5). Seventy-one spectral points were selected in the range of 300–370 nm at a 1 nm interval. Then, the spectral data were exported to MATLAB for data manipulation. The resulting data matrix (25 × 71) had 25 rows (25 mixtures) and 71 columns (spectral wavelength points). Wavelengths greater than 370 nm had absorbance values close to zero and the wavelengths lower than 300 suffered from noise.

#### 2.1.2. CLS Model Construction

The absorbance matrix of the twelve samples of the training set (12 × 71) and their corresponding concentration matrix (12 × 3) were used to construct the absorptivity matrix of the CLS model.

#### 2.1.3. PCR and PLS Model Construction

**Figure 3:**
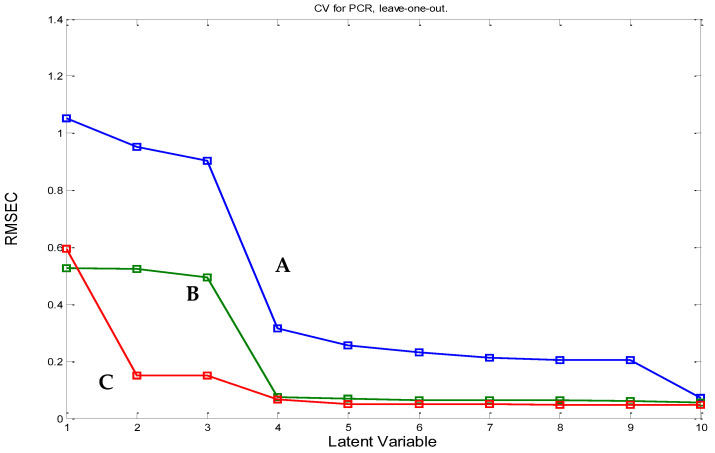
The root mean square error of the calibration (RMSEC) plot of the results of the cross-validation of the training set in the PCR model of (A) FRV, (B) Impurity-1, and (C) Impurity-2.

The raw and full spectra without using any pre-steps for data processing were utilized for the development of the PCR and PLS models where the cross validation tool (leave-one-out) was utilized to obtain the optimum number of latent variables (LV), depending on leaving-out-one sample each time using the twelve training set spectra [34]. Four latent variables were found to be the optimum number of latent factors based on having the lowest prediction error value in both the PCR and PLS models (Figure 3). The increasing number of LVs was due to the spectral and chemical similarity between FVR and the studied impurities, leading to the inaccurate determination of the exact component number in the mixture.

#### 2.1.4. GA-PLS Model Construction

PLS is a technique that uses full spectral points. Its performance and predictive ability can be improved using wavelength selection before the calibration [35]. John Holland introduced genetic algorithms (GA) as an excellent selection technique [36]. The GA technique was performed on the PLS model to select the optimum number of wavelengths that can improve the prediction ability of the PLS model. It was found that GA reduced the matrix of the wavelengths to 45.07 % for FVR, 40.85% for Impurity-1, and 42.55 % for Impurity-2. The GA optimized parameters are illustrated in Table 1 and Supplementary Materials Figure S1.

#### 2.1.5. ANN Model Construction

The ANN is one of the artificial intelligence methods that resemble the human nervous system in the capability to find the correlation between inputs and outputs. The ANN model is strongly preferred to the traditional multivariate models (PCR and PLS) for modeling linear and nonlinear relationships between variables [37]. The ANN is composed of artificial neurons inter-connected by connections called weights. Neural networks are trained so that a particular input successfully leads to the target output. Therefore, a comparison of the output and the target was used for the ANN model adjustment until matching of the network output and the target occurred [38].

The developed ANN model was a feed-forward network model that was trained with the back propagation of errors learning algorithm. The feed-forward network is one of the most preferable ANN models due to its ability to capture complex relationships between the input and output layers. Thus, it could be successfully used for the construction of the regression model [39]. This was composed of three layers. First, an input layer of 71 neurons, which represented the number of spectral points, was fed. A second hidden layer, in which the inputs were passed to, was corrected and adjusted using their weights. The inputs were finally passed to the third outer layer (output layer), giving the outputs, which represent the predicted component concentration. The connections (weights) were passed forward between layers (from input to output layer), so was called the feed-forward ANN. The differences between the predicted concentrations (outputs) and actual concentrations (targets) were called the error. The error was then back propagated to the network once more to be minimized through the further adjustment of weights. ANN was trained several times, altering several functions in the MATLAB program until the error reached the minimum value. There was a significant decrease in the error values when the hidden neurons were chosen to be four, whereas increasing the hidden neuron number to more than four did not affect the error value. Therefore, the hidden neuron number was four with the purelin–purelin transfer function. The learning coefficient (Lc) was optimized at 0.01with a 0.1 decrease level and 100 increase level.

### 2.2. Models’ Validation, Evaluation, and Comparison

**Figure 4:**
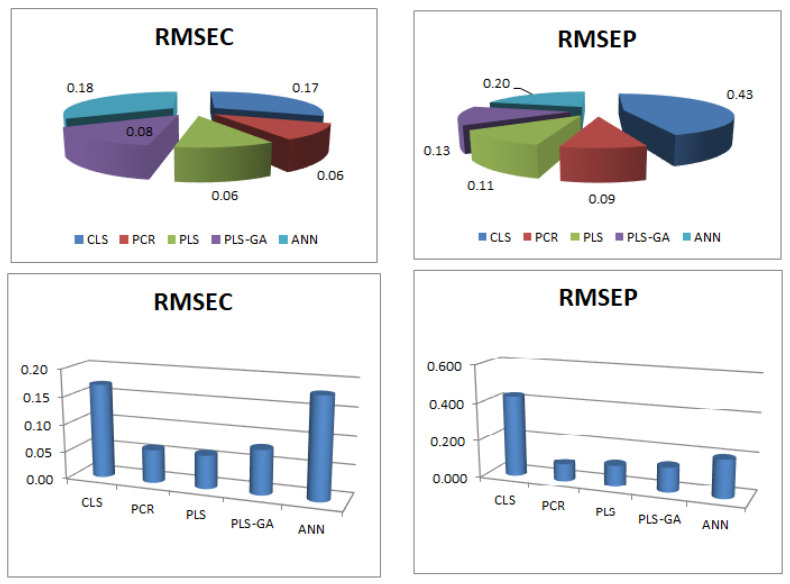
A comparison of the root mean square error of calibration (RMSEC) and the prediction (RMSEP) for FRV calculated using the different five multivariate methods (CLS, PCR, PLS, GA-PLS, and ANN).

A set of thirteen mixtures were used to validate the developed calibration models. The predictive abilities of all developed models were evaluated by calculating the predicted concentrations of the three components in the validation set mixtures, their mean recoveries, and the root mean square error of prediction (RMSEP), as illustrated in Table 2. The five models were compared as the RMSEC and RMSEP for FVR (Figure 4). Validation was also performed by plotting the theoretical concentrations against the predicted ones of the validation set mixtures and estimating the correlation coefficients (r) and slopes (Supplementary Figure S2). Additionally, graphs relating to the concentration residuals and the predicted ones were constructed (Supplementary Figure S2). The important performance parameters for the five developed models are illustrated in Table 2.

From all of the previous diagnostic tools, the predictive abilities of the five developed models were compared to evaluate the validity of each method. The RMSEC was calculated based on the results of the twelve samples of the training set and the RMSEP was calculated based on the results of the thirteen samples of the validation set, as illustrated in Table 2, with respect to the RMSEC, RMSEP, correlation coefficients (r), and percentage recoveries (Figure 4 and Table 2). The PCR and PLS models were found to have the least values of the RMSEC, RMSEP, and the highest percentage recoveries. Their correlation coefficients (r) were found to be more than 0.9990 in comparison with the other three methods which had lower (r) values. Hence, the PCR and PLS models had better linearity relationships than the other three models (Table 2).

Better agreement between the theoretical and predicted concentrations of the three components was found in the PCR and PLS models. The residual graphs indicated better random residual distribution around zero in the PCR and PLS models than in the other three models.

CLS is helpful when all of the constituents are known, but does not succeed in the presence of any degradation product or process impurities with the target compound. In contrast, PCR and PLS are very useful in the identification of the presence of a non-modeled constituent. Additionally, ANN is generally used when the models are nonlinear, which is not applicable in the present case. These reasons could explain that PCR and PLS proved to have the best performances, predictive ability, accuracy, and precision in the present case. Furthermore, PLS showed superiority regarding the sensitivity parameter.

### 2.3. Methods’ Application and Statistical Comparison

The five developed models were applied for the determination of FVR in marketed pharmaceutical dosage form (Avipiravir^®^ tablets). The results of the five models showed excellent recoveries concerning the FVR concentrations and the absence (zero concentration) of both Impurities-1 and -2 in the tablet formulation (Table 3).

The impurities under study were not detected in the Avipiravir^®^ tablets. Results for FVR obtained using the five developed methods were statistically compared with the reported method [19]. No significant difference was observed between the results (Table 3).

### 2.4. Assessment of the Proposed Method against Reported Research

The assessment of the ecological impacts of newly developed analytical methodologies has grown notably in the past few years. After the introduction of the twelve principles of green analytical chemistry (GAC), researchers raced to develop metrics that could reflect the impacts of the analytical methodologies [40]. Among those metrics, the analytical eco-scale was the first to be widely and effectively applied [41], however, this metric did not consider several steps that were involved in the analytical procedures. Therefore, in 2018, Płotka-Wasylka developed a simple green analytical procedure index (GAPI) that covered 15 steps that can occur systematically during any analytical procedure [42]. This metric covered the type of analytical methodology, sampling, sample preparation, instrumentation as well as the safety of the reagents and waste produced. The GAPI depends on a three color code, red/yellow/green, where red and green indicate high and low ecological impacts, respectively. Later, a new AGREE-tool was developed by her colleagues in the same university [43]. AGREE was focused on the twelve principles of GAC to establish a clock-shaped pictogram colored with the same three colors suggested by GAPI. However, it added a fraction numerical estimation for the greenness assessment to simplify the procedure of comparison. A value of “1” represents the highest ecological safety, which decreases upon increasing the method’s ecological impact.

Table 4 summarizes the points of comparison between the proposed method and the only reported method that considered FVR impurities [30]. The developed method covered lower ranges for the drug as well as its PRIs, while the reported method covered the FVR degradation impurities. From the GAPI assessment, the developed method showed fewer red zones and more green zones than the reported methodology. The proposed UV spectroscopy used a lower amount of solvents and did not use acetonitrile (ACN), which is more ecologically persistent and harmful [40]. The energy required in the operation of UV spectroscopy as well as the complexity of operations is much less than HPLC and even more in cost savings. The AGREE assessment (Table 4) revealed better ecological safety for the proposed method, as indicated by the calculated greenness scale. More green zones representing the twelve principles of GAC are shown in the colored pictogram.

Apart from being the first to be develop the methodology reported for the quantification of FVR and its PRIs, the proposed method was evaluated on the GAPI and AGREE metrics in order to show its ranking among some of the already reported methodologies for the determination of FVR. When comparing the performances, in addition to the excellent selectivity of the developed models to determine the three components simultaneously, they have the advantage of shorter analysis time, minimal solvent consumption, and lower cost compared to the reported HPLC methods. The developed models surpassed the chromatographic methods due to its simplicity, cost effectiveness, success of direct determination without preprocessing, and time saving. The multi-level multi-factor designs were optimized regarding the spectral range and multi-factor composition. The evaluation of the developed models’ performances in the impurity profiling of FVR was performed according to the root mean square error of calibration (RMSEC) and prediction (RMSEP), correlation coefficients (r), and percentage recoveries.

## 3. Materials and Methods

### 3.1. Materials

Pure analytical grades of FVR (Purity 99.8%), Impurity-1 (6-chloro-3-hydroxypyrazine-2-carboxamide), and Impurity-2 (3,6-dichloropyrazine-2-carbonitrile) were supplied by the Egyptian International Pharmaceutical Industries Co. (EIPICo., Tenth of Ramadan city, Egypt). Methanol, HPLC grade, was purchased from Fischer Scientific, UK. Thee Avipiravir^®^ tablets (batch number: 2008230) were supplied by EVA Pharma Co., Cairo, Egypt. Each tablet was labeled to contain 200 mg FVR.

### 3.2. Instruments and Software

A Schimadzu double beam spectrophotometer (model UV-1201, Kyoto, Japan) was equipped with 1 cm quartz cells and connected to a PC computer loaded with UV probe software version 2.43. All chemometric models were performed using MATLAB 8.2.0.701 (R2013b). The PLS and GA-PLS models were performed using PLS-toolbox software. The ANN was performed using the Neural Network Toolbox™ built in MATLAB.

### 3.3. Standard Stock Solutions

Standard stock solutions of FVR, Impurity-1, and Impurity-2 were prepared individually at concentrations of 100 µg mL^−1^ by dissolving 10 mg of each compound in methanol using 100 mL volumetric flasks. Stock and working standard solutions were found to be stable for four days in a refrigerator (4–8 °C).

### 3.4. Spectral Characteristics

The absorption spectra of 20 µg mL^−1^ FVR, 3 µg mL^−1^ Impurity-1, and 3 µg mL^−1^ Impurity-2 were scanned against methanol as the blank in the range of 200–400 nm.

### 3.5. Multi-Level Multi-Factor Design Construction

The step of the construction of the calibration and validation sets was based on multi-level multi-factor design [44]. Five concentration levels of each compound were used to construct a five-level, three-factor chemometric design, giving rise to twenty-five mixtures. The concentration levels were chosen according to the linearity range of the target compounds. The zero level of the design was 20, 3, and 3 µg mL^−1^ for FVR, Impurity-1, and Impurity-2, respectively. The design confirmed that each compound would be measured five times at each concentration from the five levels. Twelve mixtures were utilized for the construction of the calibration set and the other thirteen mixtures were utilized as a validation set to test the predictive ability of the developed models (Table 5). The twenty-five mixtures were prepared by transferring different aliquots of the target compound solutions into a series of 10 mL volumetric flasks. The volume was completed to 10 mL with methanol. The mixture solutions were scanned from 300 to 370 nm with a 1 nm interval against methanol as a blank. The ASCII data-files of the scanned spectra were saved using UV-probe software.

The construction of the calibration models of the CLS, PCR, PLS, GA-PLS, and ANN methods was performed by feeding the MATLAB software with the absorbance data and concentrations. The optimized calibration model was applied to the samples’ spectra and the concentration of each compound in the mixtures was calculated.

### 3.6. Preparation of Dosage Form for Analysis

Five tablets were accurately weighed, pulverized, and mixed well. In a 100 mL volumetric flask, a quantity equivalent to 100 mg FVR was transferred. The flask was completed to 100 mL with methanol, sonicated for 10 min, and filtered. In another 100 mL volumetric flask, 10 mL of the filtrate was diluted (1:10) with methanol to give a final concentration of 100 µg mL^−1^ FVR. Different aliquots of the diluted extract were transferred into a series of 10 mL volumetric flasks, diluted to 10 mL with methanol and scanned from 300 to 370 nm with a 1 nm interval against methanol as a blank.

## 4. Conclusions

Five chemometric models (CLS, PCR, PLS, GA-PLS, and ANN) were applied for the quantitative determination of favipiravir (T-705) and its two process related impurities. The developed models were applied for direct manipulation of the spectroscopic data. The PCR and PLS models can be used successfully for the impurity profiling of favipiravir due to their powerful resolving abilities without the need of separation or an extraction pre-step. Therefore, they could be alternatives to the costly liquid chromatographic techniques with a faster analysis time. A comparative study was performed between the five developed models. PCR and PLS proved to be the best models regarding their performances and predictive abilities. PLS was found to have better sensitivity than PCR and the other three models. The proposed methods’ greenness was evaluated on the GAPI and AGREE metrics, which showed a more ecofriendly approach.

## Figures and Tables

**Table 1:** The genetic algorithm parameters.

Parameter	FVR	Impurity-1	Impurity-2
Population size	36	36	36
Maximum generations	49	49	49
Mutation rate	0.005	0.005	0.005
The number of variables in a window (window width)	2	2	2
% population the same at convergence	100	100	100
% Wavelengths used at initiation	50	50	50
Crossover type	Double	Double	Double
Maximum number of latent variables	6	6	6
Cross validation	Random	Random	Random
Number of subsets to divide data into for cross validation	5	5	5

**Table 2:** The performance parameters of the five developed models for FVR determination.

Parameter	CLS	PCR	PLS	GA-PLS	ANN
Wavelength range *	300–370 nm				
RMSEC	0.17	0.06	0.06	0.08	0.18
RMSEP	0.43	0.09	0.11	0.13	0.2
LV number	-	4	4	4	-
Mean (%) **	98.64	99.99	99.69	99.08	100.82
R	0.7543	0.9994	0.9995	0.9990	0.9989

* Selection by the trial and error method based on the RMSEC. ** Calculated for the prediction set.

**Table 3:** The recovery results and statistical comparison between the results of FVR determination obtained by the proposed methods and the reported method [19] in the Avipiravir^®^ tablets.

Parameter	Claimed Conc. of FVR in Tab (µg mL^−1^)	Percentage Recovery (%)				
		CLS	PCR	PLS	GA-PLS	ANN
	18	99.91	100.73	101.70	100.55	100.22
	19	98.88	99.93	100.74	99.64	100.84
	20	100.00	100.65	102.55	99.87	101.17
	21	98.19	101.03	101.74	100.57	99.98
	Reported method ^a^ [19]	CLS	PCR	PLS	GA-PLS	ANN
Mean ± SD	100.80 ± 1.50	99.25 ± 0.87	100.58 ± 0.47	101.68 ± 0.74	100.16 ± 0.47	100.55 ± 0.55
V	2.25	0.75	0.22	0.55	0.22	0.30
N	3	4	4	4	4	4
F-test (F-tabulated 28.71) ^b^	--	3.00	10.23	4.09	10.23	7.50
Student’s *t*-test (*t*-tabulated 2.571) ^b^	--	1.747	0.284	1.039	0.825	0.315

^a^ The spectrofluorometric method based on the determination of FVR in Britton–Robinson buffer at pH 4 at 436 nm as the emission wavelength and 323 nm as the excitation wavelength. ^b^ Figures in parentheses are the corresponding tabulated values at *p* = 0.05.

**Table 4:** A comparison of the proposed analytical methods for the chosen reported methodologies.

Parameters	Proposed Spectroscopic Method	Reported LC Method [30]
Technique	UV Spectroscopy	Ion pair HPLC-DAD
Linearity range	2.0–20.0 µgmL^−1^	6.3–250.0 µgmL^−1^
Impurity profile	Process related impurities	Degradation impurities
Organic solvents	Methanol as the solvent	Methanol as the solvent. Isocratic elution using 28% MeOH & 10% ACN
Run time	NA	5 min
Flow rate	NA	1.0 mL min^−1^
Column	NA	C18-RP
GAPI assessment *	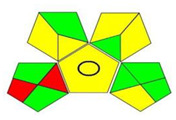	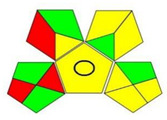
AGREE assessment *	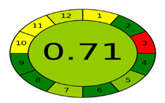	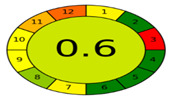

* According to the used greenness metrics, the ecological impact ranges from highest (red color), medium (yellow color), to lowest (green color).

**Table 5:** The concentrations of FVR and the impurities in the laboratory prepared mixtures used in the calibration and validation sets.

Sample No.	FVR (μg mL^−1^)	Impurity-1 (μg mL^−1^)	Impurity-2 (μg mL^−1^)
1 *	20	3	3
2 *	20	2	2
3	19	2	4
4 *	19	4	2.5
5	21	2.5	4
6 *	19.5	4	3
7	21	3	2.5
8 *	20	2.5	2.5
9 *	19.5	2.5	3.5
10 *	19.5	3.5	4
11 *	20.5	4	3.5
12 *	21	3.5	3
13	20.5	3	4
14 *	20	4	4
15	21	4	2
16	21	2	3.5
17	19	3.5	2
18	20.5	2	3
19	19	3	3.5
20	20	3.5	3.5
21 *	20.5	3.5	2.5
22 *	20.5	2.5	2
23	19.5	2	2.5
24	19	2.5	3
25 *	19.5	3	2

* These mixtures were used in the validation set.

## Data Availability

All data are available from the corresponding author upon request.

## Supplementary Materials

The following supporting information can be downloaded at: https://www.mdpi.com/article/10.3390/molecules27123658/s1, Supplementary Figure S1: Parameters involved in the GA-PLS model for FVR, Supplementary Figure S2: Plot of the actual FVR concentration versus (A) predicted concentrations, (B) residual concentrations in the ANN model.

## Sample Availability

Samples of the compounds are not available from the authors.
