# Supplementary material for: Analytical Performance and Greenness Evaluation of Five Multi-Level Design Models Utilized for Impurity Profiling of Favipiravir, a Promising COVID-19 Antiviral Drug
Source: Molecules. 2022 Jun 7;27(12):3658. doi: 10.3390/molecules27123658 (PMC9229086; doi:10.3390/molecules27123658)
Supplement: Supplementary file 1 [file molecules-27-03658-s001.zip › molecules-1741609-supplementary.pdf]

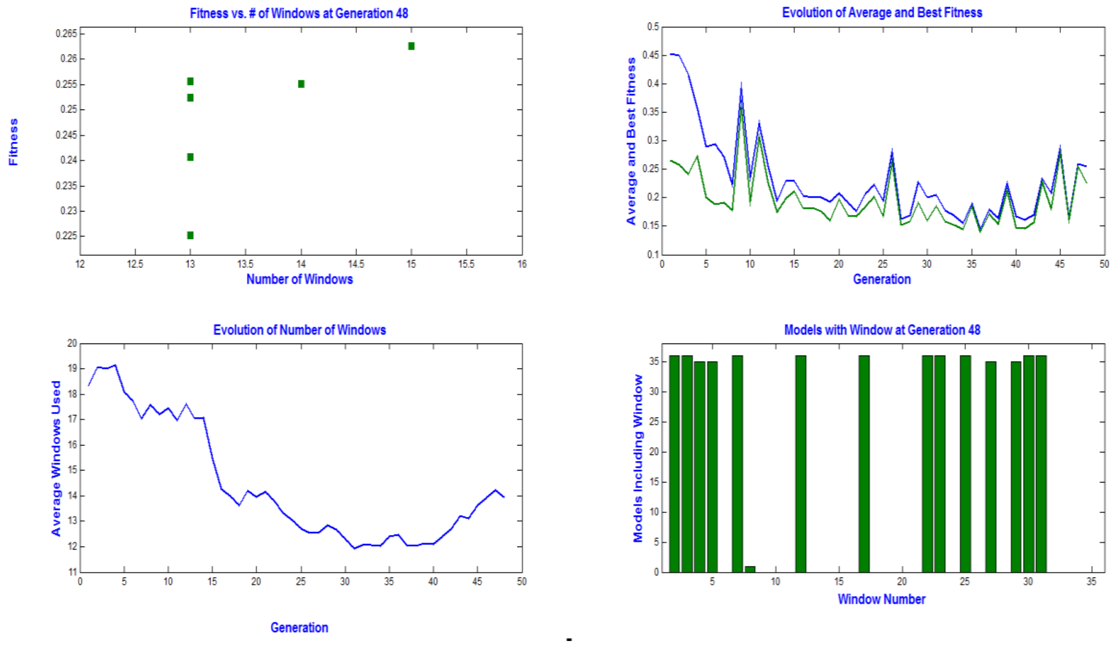

Supplementary Figure S1. Parameters involved in GA-PLS model for FVR

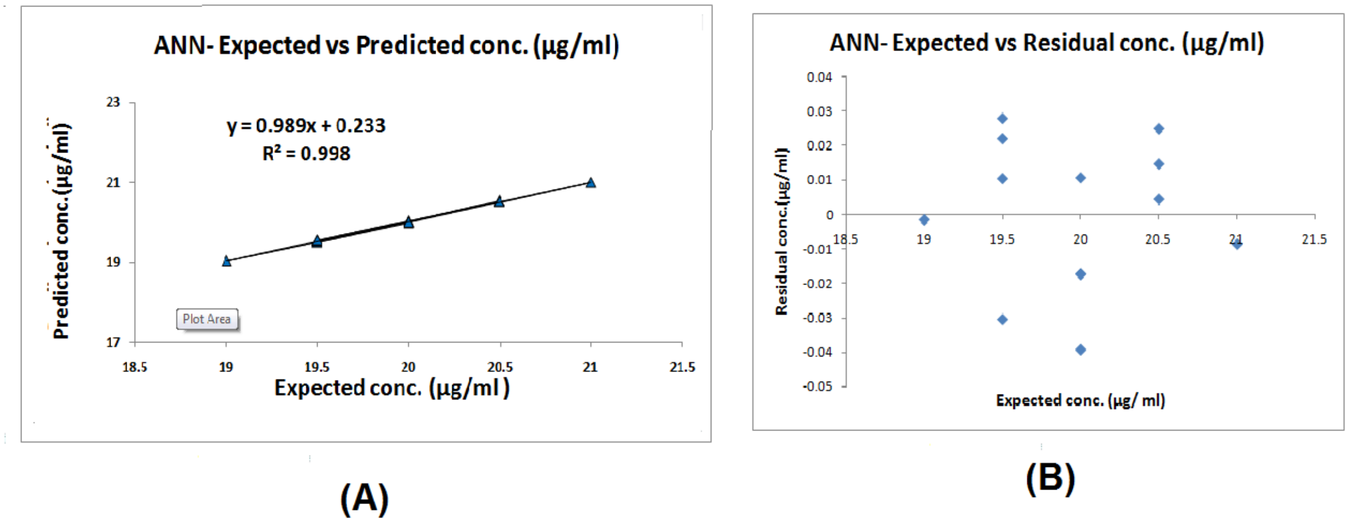

Supplementary Figure S2. Plot of actual FVR concentration versus (**A**) predicted concentrations, (**B**) residual concentrations in ANN model
